# Supplementary material for: Detection of low-density Plasmodium falciparum infections using amplicon deep sequencing
Source: Malar J. 2019 Jul 1;18:219. doi: 10.1186/s12936-019-2856-1 (PMC6604269; doi:10.1186/s12936-019-2856-1)
Supplement: Supplementary file 1 — Additional file 1. Additional tables and figures. [file 12936_2019_2856_MOESM1_ESM.pdf]

Additional File 1: Tables and Figures

Early *et al*., Detection of low-density *Plasmodium falciparum* infections using amplicon deep sequencing

Table S1: Strain composition of mock *Plasmodium*/human DNA mixtures

Total genome copies/ul	Strain Proportion					
	3d7	Mcamp	Th029.09	Th002.09	Th135.09	Dd2
1.34	0	1	0	0	0	0
2.18	0	1	0	0	0	0
2.21	0	0	0	0.04	0.96	0
2.65	0.89	0.11	0	0	0	0
2.98	0	0	0	0.52	0.48	0
3.02	0	1	0	0	0	0
3.47	0	0	0	0.04	0.96	0
3.67	0.96	0.04	0	0	0	0
4.16	0.89	0.11	0	0	0	0
4.29	0.99	0.01	0	0	0	0
4.31	0	0	0	0	1	0
4.50	0.99	0.01	0	0	0	0
4.67	0	0	0	0.52	0.48	0
4.7	0	1	0	0	0	0
4.70	1	0	0	0	0	0
5.04	0	0	0	0.04	0.96	0
5.77	0.96	0.04	0	0	0	0
6.03	0.89	0.11	0	0	0	0
6.74	0.99	0.01	0	0	0	0
6.78	0	0	0	0	1	0
6.79	0	0	0	0.52	0.48	0
7.06	0.99	0.01	0	0	0	0
7.39	1	0	0	0	0	0
8.17	0	0	0	0.04	0.96	0
8.38	0.96	0.04	0	0	0	0
9.79	0.99	0.01	0	0	0	0
9.79	0.89	0.11	0	0	0	0
9.84	0	0	0	0	1	0
10.03	0	0.19	0	0	0.81	0
10.26	0.99	0.01	0	0	0	0
10.73	1	0	0	0	0	0
11.01	0	0	0	0.52	0.48	0
11.30	0	0	0	0.04	0.96	0
13.55	0.89	0.11	0	0	0	0
13.60	0.96	0.04	0	0	0	0
14.75	0.33	0.04	0	0.33	0.30	0
14.88	0.32	0.04	0.04	0.32	0.29	0

15.23	0	0	0	0.52	0.48	0
15.88	0.99	0.01	0	0	0	0
15.96	0	0	0	0	1	0
16.64	0.99	0.01	0	0	0	0
17.4	0	0	0	1	0	0
17.4	1	0	0	0	0	0
17.61	0	0	0	0.04	0.96	0
18.81	0.96	0.04	0	0	0	0
20.07	0	0.19	0	0	0.81	0
20.95	0.32	0.04	0.04	0.32	0.29	0
21.1	0.89	0.11	0	0	0	0
21.92	0	0.07	0	0	0.93	0
21.97	0.99	0.01	0	0	0	0
22.08	0	0	0	0	1	0
22.7	0.33	0.04	0	0.33	0.30	0
23.02	0.99	0.01	0	0	0	0
23.73	0	0	0	0.52	0.48	0
24.08	0	0	0	1	0	0
24.08	1	0	0	0	0	0
28.53	0.32	0.04	0.04	0.32	0.29	0
29.3	0.96	0.04	0	0	0	0
32.63	0.33	0.04	0	0.33	0.30	0
34.22	0.99	0.01	0	0	0	0
34.4	0	0	0	0	1	0
35.86	0.99	0.01	0	0	0	0
37.5	0	0	0	1	0	0
37.5	1	0	0	0	0	0
41.73	0	0.03	0	0	0.97	0
43.7	0.32	0.04	0.04	0.32	0.29	0
43.84	0	0.07	0	0	0.93	0
52.5	0.33	0.04	0	0.33	0.30	0
58.87	0.32	0.04	0.04	0.32	0.29	0
72.37	0.33	0.04	0	0.33	0.30	0
83.46	0	0.03	0	0	0.97	0
89.2	0.32	0.04	0.04	0.32	0.29	0
112.1	0.33	0.04	0	0.33	0.30	0
200	0	0	0	0	0	1

Table S2. Nucleotide differences between culture-adapted parasite lines within the *CSP* (top) and *SERA2* (bottom) amplicons

	3D7	Mcamp	Dd2	Th029.09	Th002.09	Th135.09
**3D7**	-	7	5	8	7	8
**Mcamp**	4	-	5	5	2	5
**Dd2**	3	1	-	3	5	3
**Th029.09**	1	3	2	-	6	0
**Th002.09**	4	2	1	3	-	6
**Th135.09**	4	2	1	3	0	-

A.

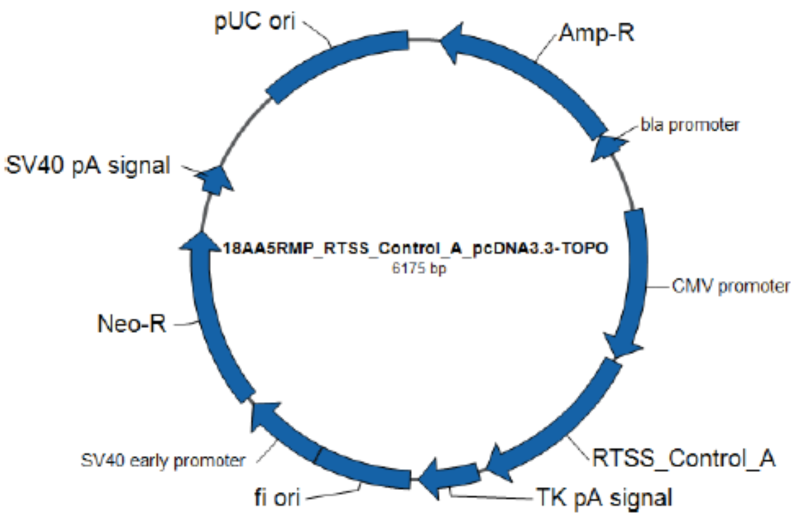

B.

GGATCCAAACTAAGATGTGTTCTTTATCTAA**TTAAGGAACAAGAAGGATAATACCA**TTATTAAT
CCTATTGAACTATCGATTACATTAAACACACTGGAACATTTTTCCATTTTACAAATTTTTTTT
CAATTCTTAATGCATAATCTAATTCGTCTTTAGGTTTATTAGCAGAGCCAGGCTTTATATCTCA
TTGAATACCATTTCCACAAGTTACACTACATGGGGACCGACTTATTGAAAGAGAATTTTGTATT
TTGTTTAAATATTCTTTTAGCTTGTTATCACTTGGTTCTTCGTTATTATTATTTTTTACAGCAC
TGTTGGCATTAGCATTTTCATCTA**CATTTCGGTTTGGGTCATTT**GGCATATTGTGACCTTGTCC
ATTACGGATCCTACAACTCCTCGAGGTAATCGTGGTAATTGTGGTCCTGC**TACTTTCCCTTGCC**
CTTGTGATCCAGGTGATATCTTTGTTCCTACGGATCCGTTCCTTCCTGATGATCTTCCTGCTCT
TTCGACATCTGATTGGGATACTTCTGCACCTGGTCTTGCTGATTCTACTTTTTGTTCTGCTCCT
ATACCACTTCCTCTTCATTTTTGTTGTTGCTGTTGTTGGGGTTGTGTTTCTTGAGCTAAAGTTA
GATGTGTTGGTTGTTGTTTTGGTTTTTCAATTCTTGCTTGTGTCTATGTACGTGAACCATCGGA
TGATAATGA**TCCTGTAGCAGATTCATCTGTAGTG**GTTTCACTCTTTATTGTATTTTTACTCGAG

Figure S1: Control plasmid map (A) and sequence (B). The order of sequence in the plasmid is: Bam-25nt_linker-CSP_sequence-25nt_linker-Bam-8nt_linker-Xho-25nt_linker-SERA2_sequence-25nt_linker-Xho. Restriction sites are highlighted in yellow. Linker sequences are highlighted in grey. The primer sequences are marked in bold letters. Colored nucleotides denote naturally occurring SNPs observed in Neafsey *et al* ([1]; blue), the Pf3k database (www.malariagen.net/projects/pf3k; orange), or both (red). Underlined nucleotides mark regions of the plasmid sequence that differ from any previously observed natural sequences. Reference sequence nucleotide content was maintained within these regions, but the order of nucleotides were shuffled.

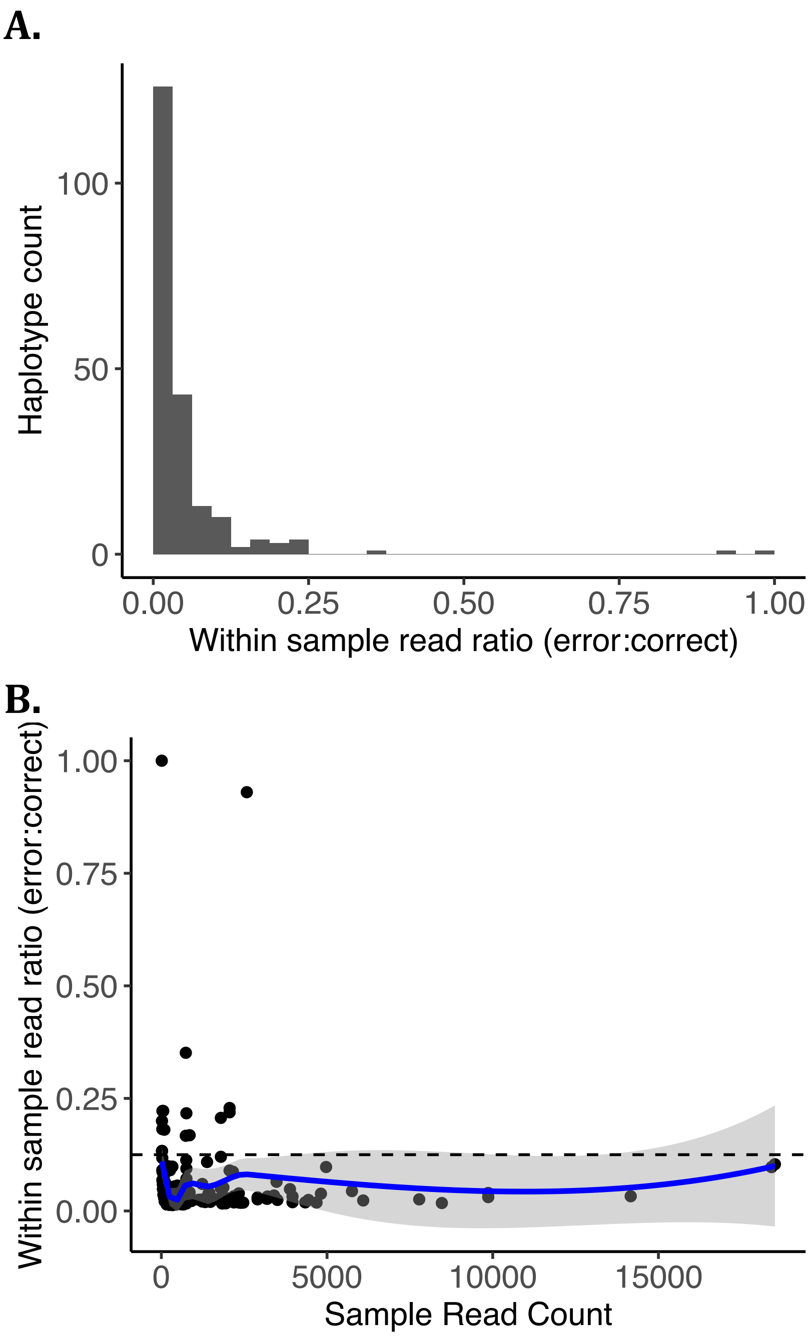

Figure S2: Relative read support for haplotypes likely resulting from PCR error. Across all mock sample sequences, 208 erroneous haplotypes both (1) differed from a true haplotype within the same sample by a single nucleotide change, and (2) appeared only once in the sequencing run. The ratio of their read support to the read count of the true haplotype is plotted. (A) 92% of erroneous haplotypes had less than 1/8 the support of the true haplotype. (B) Read ratio does not vary substantially with sample read depth. A Loess curve is marked in blue. The dashed line marks the 1:8 ratio used as a cutoff in the analyses in the manuscript.

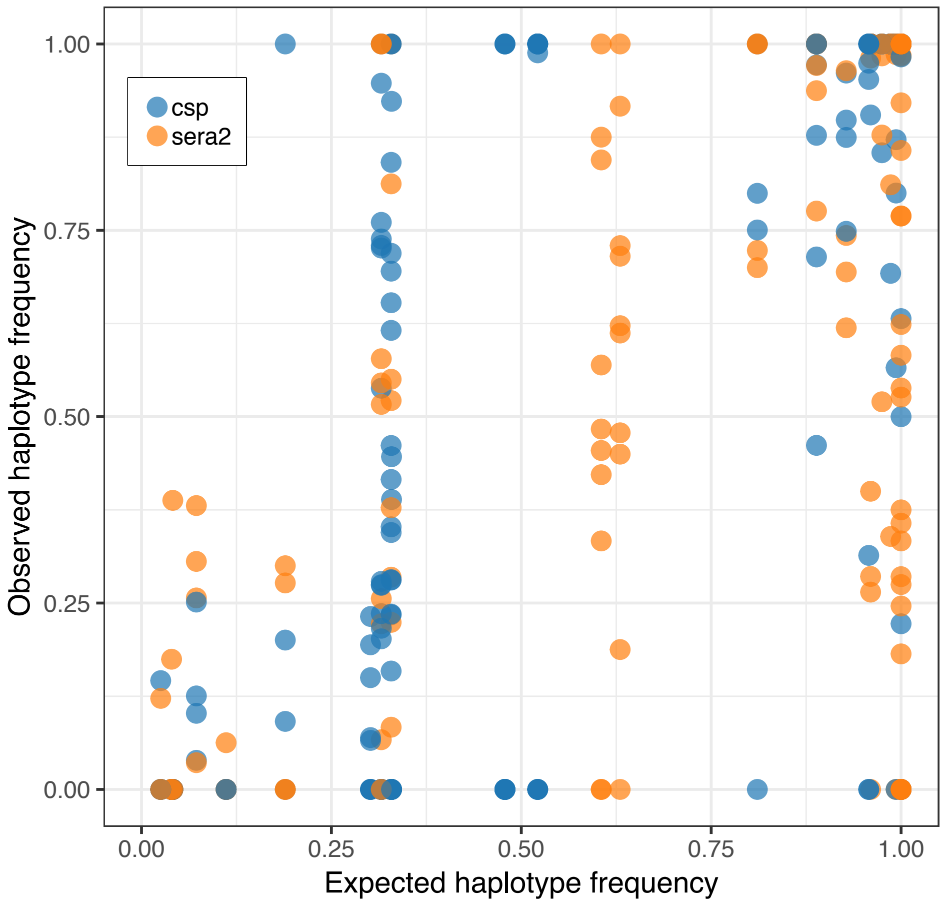

Figure S3: Expected haplotype frequency versus observed haplotype frequency within samples. Only samples with at least 100 reads are shown. Overall correlation (Pearson's r) is 0.82 ($P < 0.0001$). For haplotypes with an expected frequency between 0.1 and 0.9 is r = 0.60 ($P < 0.0001$).

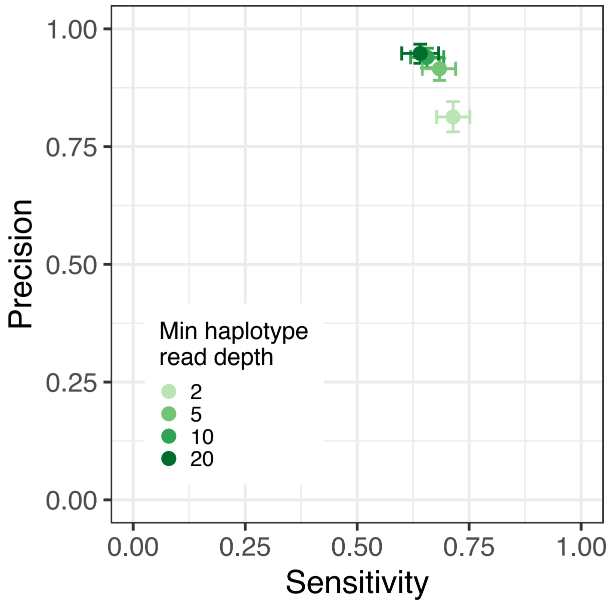

Figure S4. Precision and Sensitivity of PASEC under alternative minimum haplotype read depth thresholds.

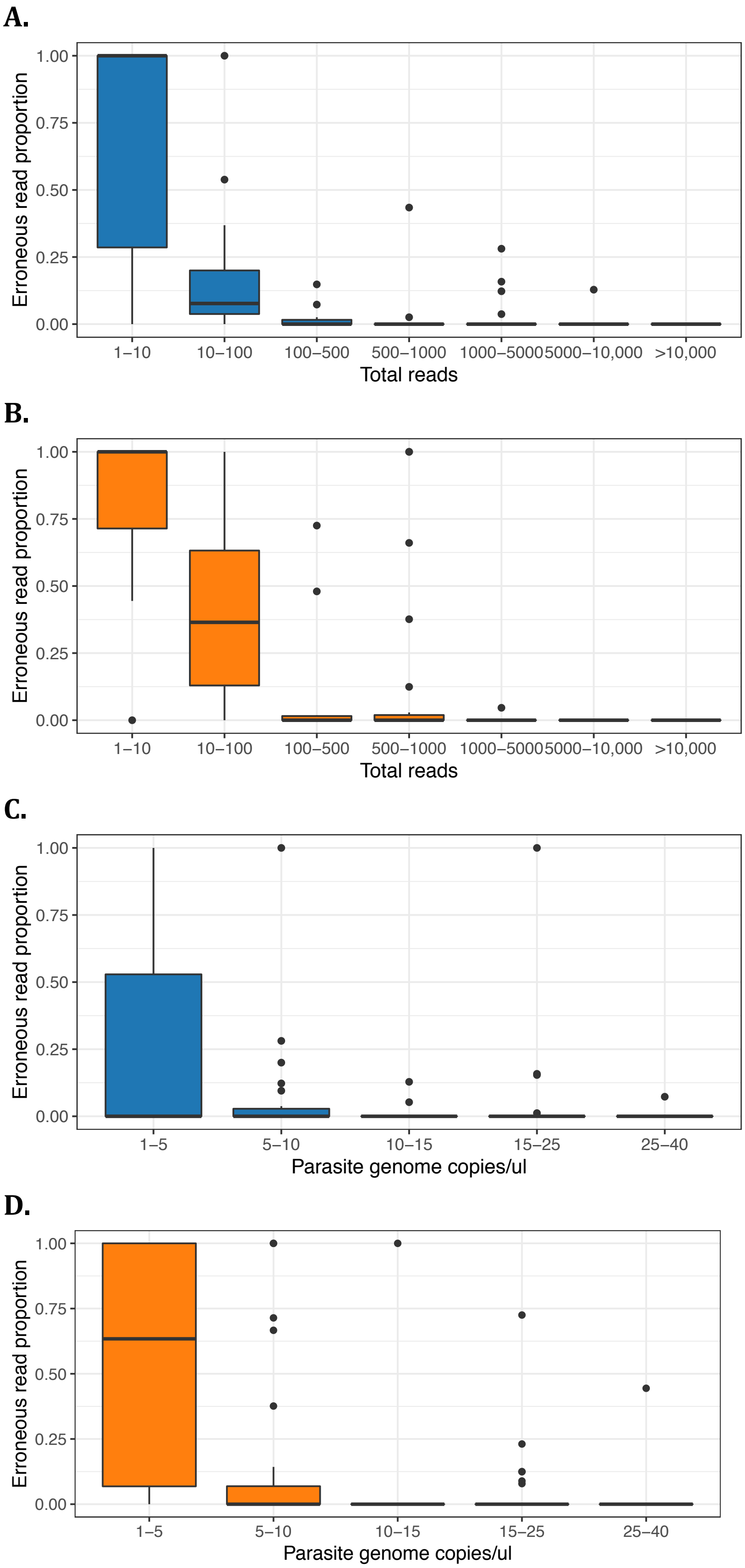

Figure S5: Parasite DNA concentration and sample read depth affect the proportion of reads that are erroneous. Samples with low read depth (A,B) and low parasite DNA concentrations (C,D) contain a higher proportion of reads that support erroneous haplotypes. Results are similar for the *CSP* (blue; A,C) and *SERA2* (orange; B,D) amplicons.

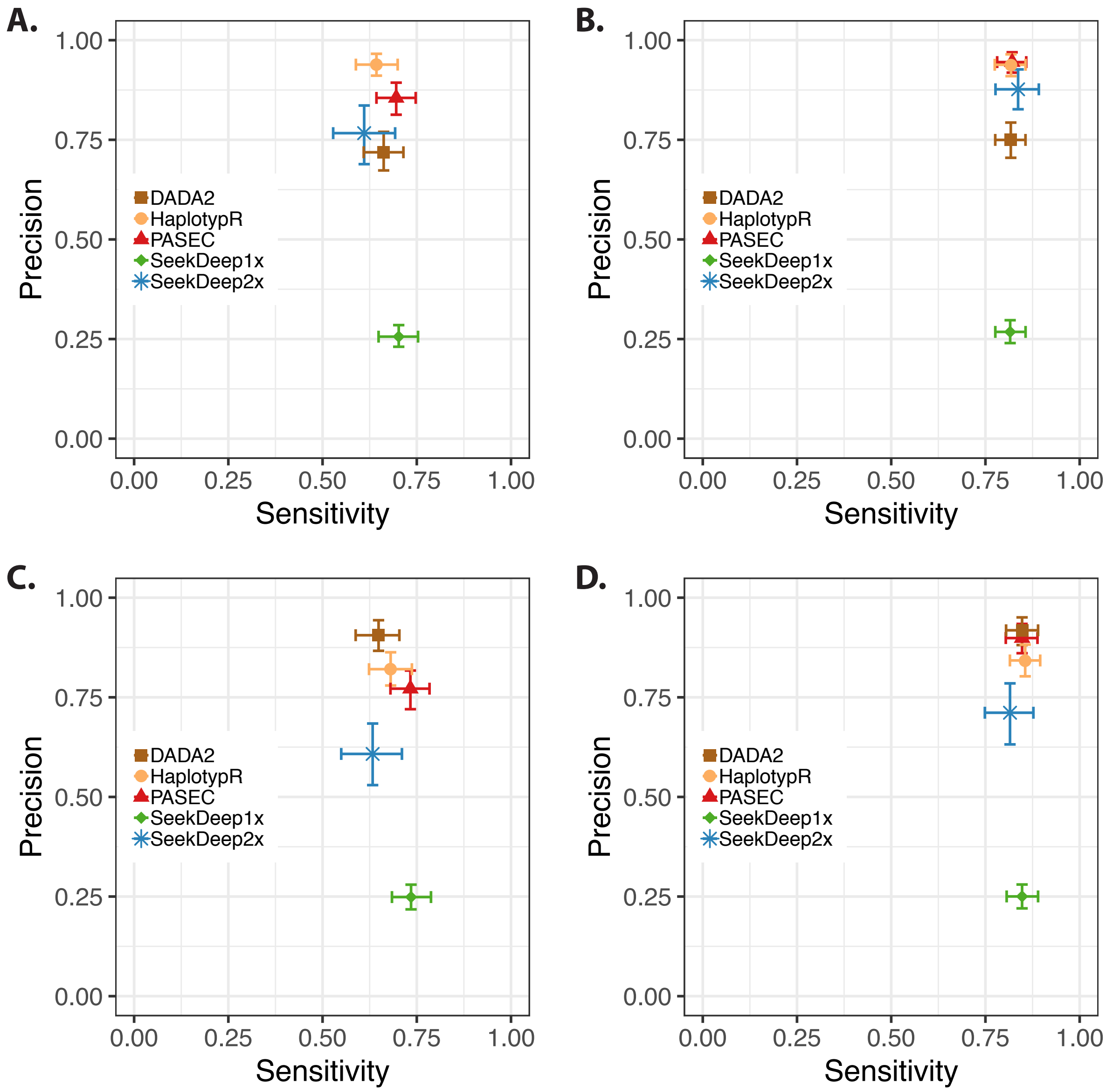

Figure S6: Sensitivity and precision of the five analysis pipelines on a per amplicon basis. Sensitivity and precision are plotted for *CSP* (A, B) and *SERA2* (C, D) The calculations for plots A. and C. were made using all samples, while plots B. and D. used only samples with at least 100 reads. 95% confidence intervals were calculated with 1000 bootstrap replicates.

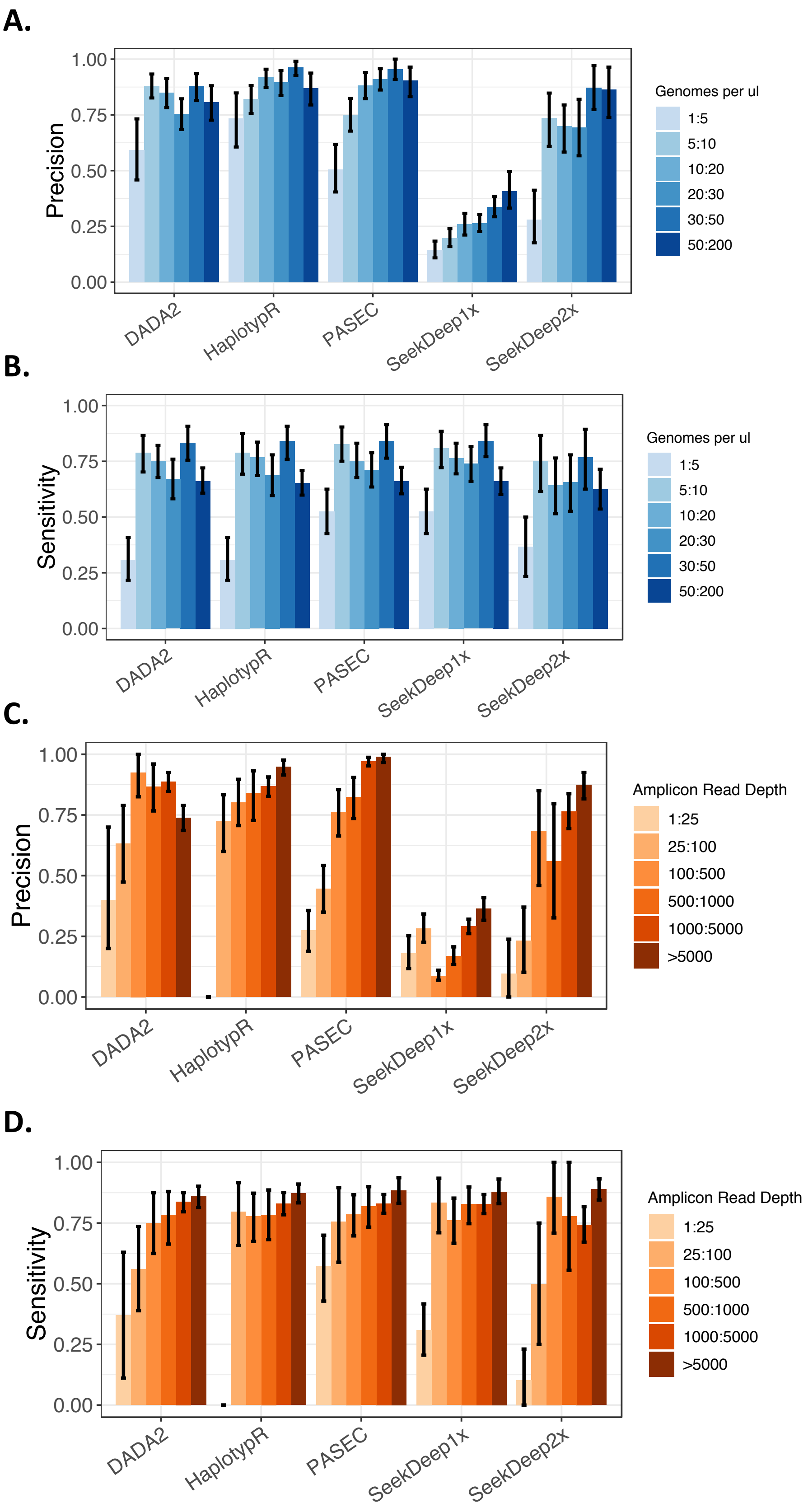

Figure S7: Precision and Sensitivity are reduced at low genome copy number and low read depths. HaplotypR best practices filter out all samples with fewer than 25 reads, so no data were available for this bin.

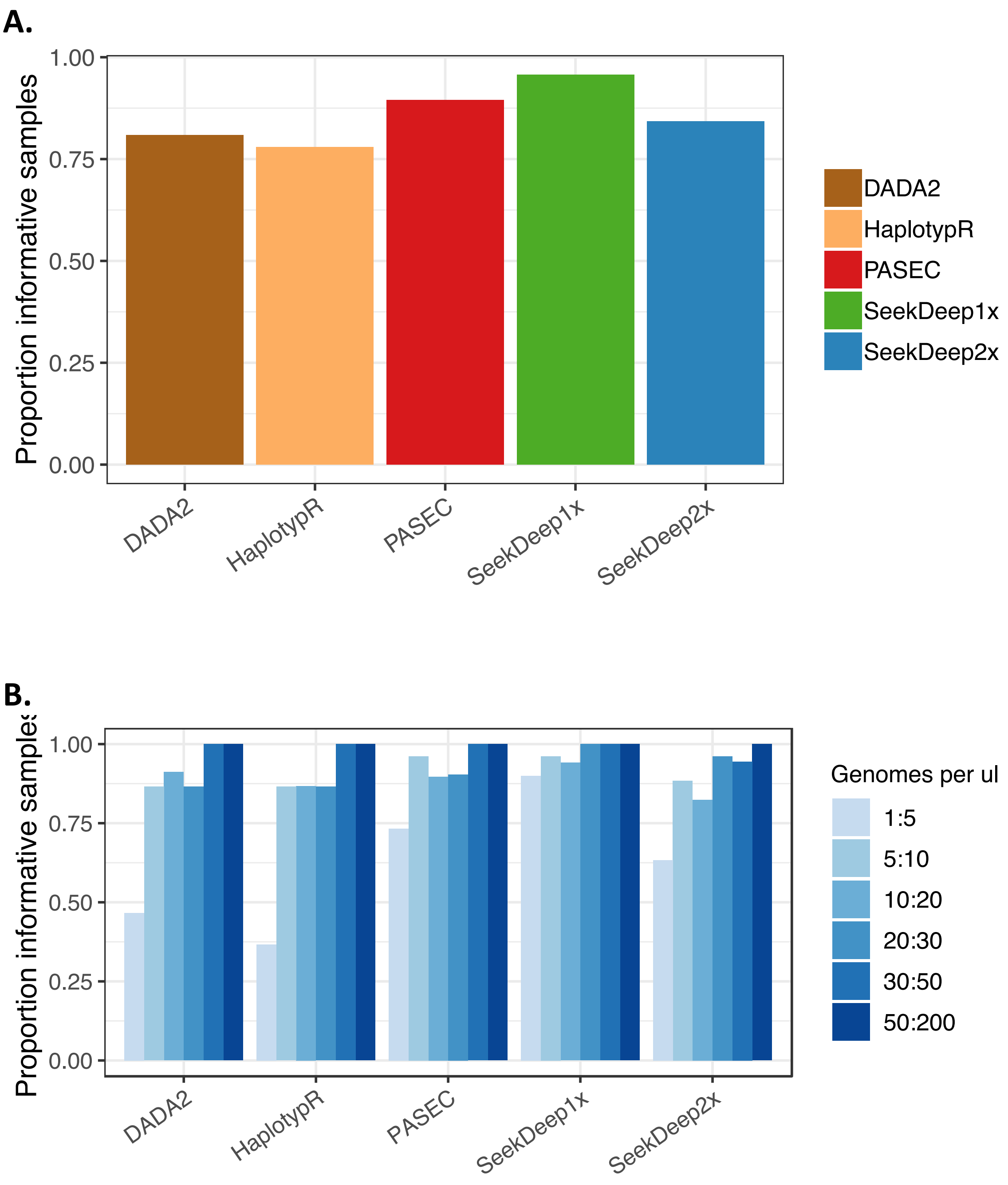

Figure S8: Proportion of samples in which haplotypes were identified by each tool. (A) Tools varied in their overall rate of sample success (the proportion of samples in which at least one haplotype was resolved). (B) Sample success was lowest for samples with low *Plasmodium* genome concentrations.

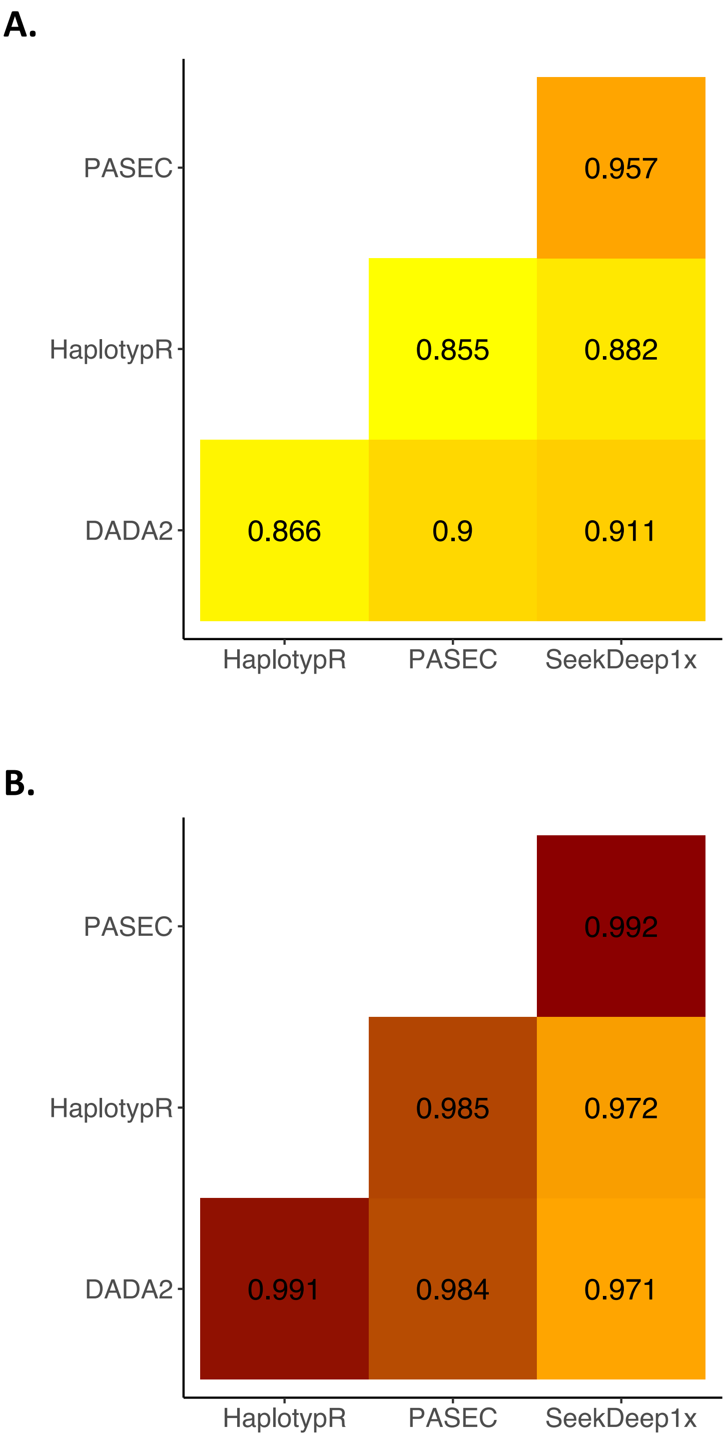

Figure S9. Correlation between tool estimates of the major haplotype frequency within mock infections. Pearson's r estimates are given for (A) all samples and (B) samples with at least 100 reads.

A.

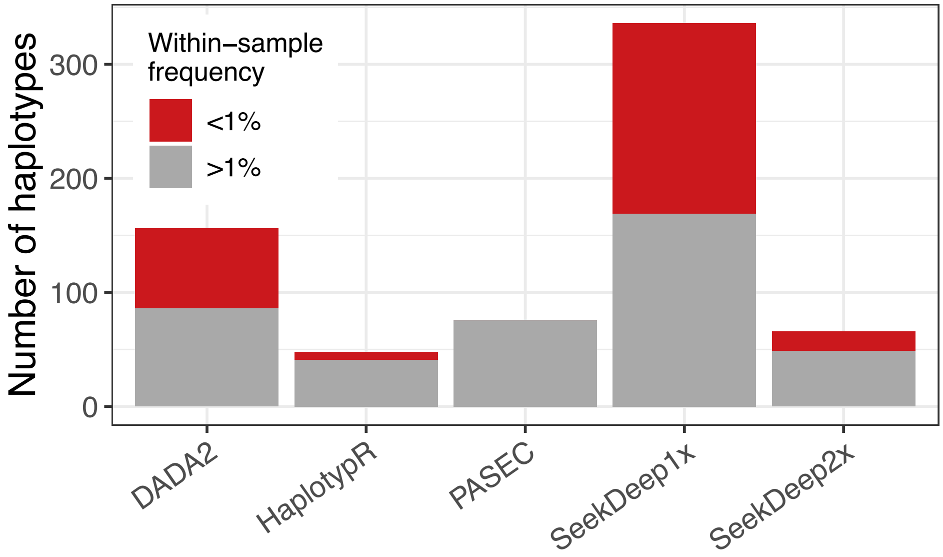

B.

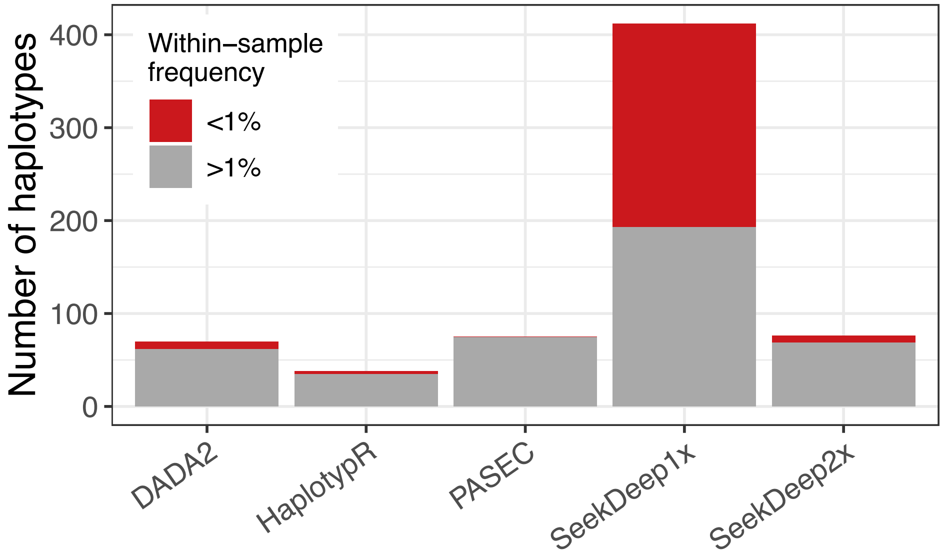

Figure S10: Number of unique haplotypes identified by each tool in a set of 190 patient samples from sub-Saharan Africa. Counts are shown for (A) *CSP* and (B) *SERA2*.

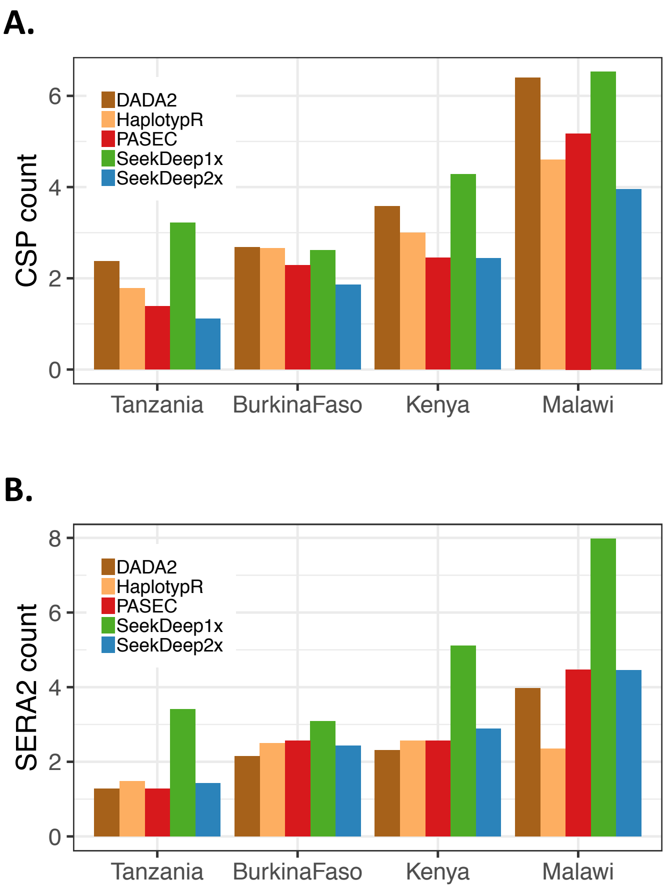

Figure S11: Average number of (A) *CSP* and (B) *SERA2* haplotypes per sample calculated by the five pipelines.

References

1. Neafsey DE, Juraska M, Bedford T, Benkeser D, Valim C, Griggs A, et al. Genetic Diversity and Protective Efficacy of the RTS,S/AS01 Malaria Vaccine. N Engl J Med. 2015;373:2025–37. doi:10.1056/NEJMoa1505819.
